# Selection of Fructophilic Yeast from Sun-Dried Pedro Ximénez Grape Must for the Development of New Vinegars Containing Gluconic Acid

**DOI:** 10.3390/foods14142410

**Published:** 2025-07-08

**Authors:** Juan Carbonero-Pacheco, Álvaro García-Jiménez, Juan C. Mauricio, Juan C. García-García, Juan J. Román-Camacho, Elena García-Muñoz, Inés M. Santos-Dueñas, Teresa García-Martínez, Isidoro García-García

**Affiliations:** 1 Department of Agricultural Chemistry, Edaphology and Microbiology, Agrifood Campus of International Excellence CeiA3, Universidad de Córdoba, 14014 Córdoba, Spain; b12capaj@uco.es (J.C.-P.); b82gajia@uco.es (Á.G.-J.); p22gagaj@uco.es (J.C.G.-G.); b32rocaj@uco.es (J.J.R.-C.); elenagarciam99@gmail.com (E.G.-M.); mi2gamam@uco.es (T.G.-M.); 2 Department of Inorganic Chemistry and Chemical Engineering, Agrifood Campus of International Excelence CeiA3, Nano Chemistry Institute (IUNAN), Universidad de Córdoba, 14014 Córdoba, Spain; ines.santos@uco.es

**Keywords:** vinegar, fructophilic yeasts, *Starmerella lactis-condensi*, osmophilic yeasts, alcoholic fermentation

## Abstract

Wine vinegar and wine are traditional Spanish products, obtained from grape must by alcoholic fermentation (wine) and subsequent acetification (vinegar). Although these are established products, there is great interest in the development of new products, particularly new vinegars, and among these, the possibility of vinegars containing gluconic acid stands out. Gluconic acid in vinegar, mainly produced by acetic acid bacteria (AAB), is positively valued by consumers. Its content depends on the availability of glucose in the base wine; however, this hexose is preferentially consumed by the indigenous yeast population which conducts the previous alcoholic fermentation. For this reason, the use of non-conventional fructophilic yeasts, which consume fructose rather than glucose, is required. In this work, we isolated, screened, and identified osmophilic and fructophilic non-*Saccharomyces* yeasts from sun-dried grape must and tested them under different fermentation conditions in synthetic and natural grape musts, in order to obtain a base wine with ethanol and glucose content for the development of new vinegars containing gluconic acid. The isolate of the species *Starmerella lactis-condensi* was found to be an ideal candidate due to its fructophilic and osmophilic features, which allowed for the production of a base wine with high ethanol (11% *v*/*v*) and glucose (up to 200 g/L) content from a natural concentrated must. In fresh must, inoculation with *Starmerella lactis-condensi* resulted in faster and preferential fructose consumption over glucose compared to the control. However, both sugars were completely consumed at the end of the alcoholic fermentation; therefore, new fermentation strategies should be tested in this type of must. Furthermore, this strain could be of interest in oenology due to its high glycerol yield and low volatile acid production during alcoholic fermentation. The use of this strain could allow for the production of new wines with unique metabolic profiles suitable for further vinegar production.

## 1. Introduction

Vinegar is one of the most appreciated fermented foods, and it is widely used as a preservative, condiment, and flavoring additive by mankind [1,2]. Vinegar is mainly produced by a diverse population of acetic acid bacteria (AAB) species; these microorganisms transform the ethanol present in the medium into acetic acid. Although vinegar can originate from various substrates, in Mediterranean countries, wine vinegar derived from grape must is the most common source [2,3]. Thus, wine vinegar is produced after two fermentation processes; firstly, the alcoholic fermentation carried out mainly by yeasts, which transform the sugar of must (glucose and fructose) into ethanol, glycerol and other compounds; ethanol is later consumed by AAB, producing acetic acid during acetic fermentation [4].

AAB are Gram-negative microorganisms which are primarily responsible for the acetic fermentation process due to their oxidative capabilities. AAB diversity varies according to the substrate, with the genus *Komagataeibacter* being the most important one in quantitative terms, followed by other genera, such as *Acetobacter*, *Gluconobacter*, and *Gluconoacetobacter* [1]. This diversity also entails a wide range of metabolic pathways, especially those related to carbon source assimilation, including alcohols, sugars, and sugar alcohols. The most commonly utilized carbohydrate source is glucose; however, other carbohydrates can also be metabolized; i.e., the oxidation of glucose to glucono-δ-lactone, which is subsequently hydrolyzed to D-gluconate (gluconic acid) by the species *Gluconobacter oxydans*, has been extensively studied [5,6].

D-gluconate increases the fixed acidity of vinegar, thereby enhancing its physical stability and modifying its sensory profile, which may result in an improvement of vinegar properties [7]. One example of high gluconate vinegar is Traditional Balsamic Vinegar (TBV), which is produced from grape must that has been previously cooked to concentrate the sugar content, then a spontaneous fermentation begins, where yeast produce ethanol from available fermentable sugars and subsequently AAB assimilate it and generate acetic acid and D-gluconate [7]. Yeasts involved in this process are osmotolerant, and some are also fructophilic, features that make them well-suited for carrying out alcoholic fermentation in this substrate, tolerating high osmotic pressure and consuming fructose over glucose, thereby leaving glucose available for acetic acid bacteria which will transform the hexose in D-gluconate (AAB) [7,8]. Yeast species with these characteristics, such as *Saccharomycodes ludwigii*, *Starmerella lactis-condensi*, *Zygosaccharomyces rouxii* or *Z. bailii*, have been isolated and identified during the production process of TBV as well as other high-osmolarity products, such as Tokaj Essence wines (botrytised high-sugar wines from Hungary) [7,9]. The presence of a fructose membrane transporter designated Fructose Facilitator Zygosaccharomyces (FFZ1) is responsible for their affinity to this hexose [10,11].

In southern Spain, sweet wines called “Pedro Ximénez” with a sugar content up to 500 g/L are produced in the Montilla–Moriles Protected designation of origin (PDO) [12]. This concentration is achieved through a technique known as “asoleo”, which involves exposing the grapes to sunlight, inducing dehydration and thereby sugar concentration. Subsequently, the resulting must is fortified with wine ethanol to 15% (*v*/*v*) to prevent alcoholic fermentation, then, an aging process in oak wood barrels is carried out prior to wine commercialization [13]. Osmophilic *Saccharomyces cerevisiae* strains have been isolated from spontaneous fermentation of sun-dried grape Pedro Ximénez must; however, the presence of other yeast species or their fructophilic trait is still unknown [13]. Currently a sweet vinegar is produced within this PDO using this type of product. Its elaboration consists of blending sun-dried grape must with wine vinegar [14]. This is due to the must characteristics in which conventional yeasts are unable to carry out an alcoholic fermentation that would generate enough ethanol to use this substrate for vinegar production. Osmophilic yeast could perform the process in this type of substrate; however, they will produce ethanol through glucose consumption which would not allow for the production of D-gluconate by AAB after alcoholic fermentation. The inoculation with non-conventional fructophilic and osmophilic yeasts could resolve this issue and allow the use of sun-dried Pedro Ximénez grape must in the production of a new sweet vinegar in which ethanol is produced from fructose by yeasts and, subsequently, D-gluconate from glucose and acetic acid from ethanol by AAB metabolism as occurs in TBV [1,7].

The objective of this study is to isolate and identify osmotolerant and fructophilic yeasts from a sun-dried grape must and subsequently evaluate their potential for producing a base wine with glucose that enables the development of a new type of vinegar containing gluconic acid.

## 2. Materials and Methods

**Figure 1:**
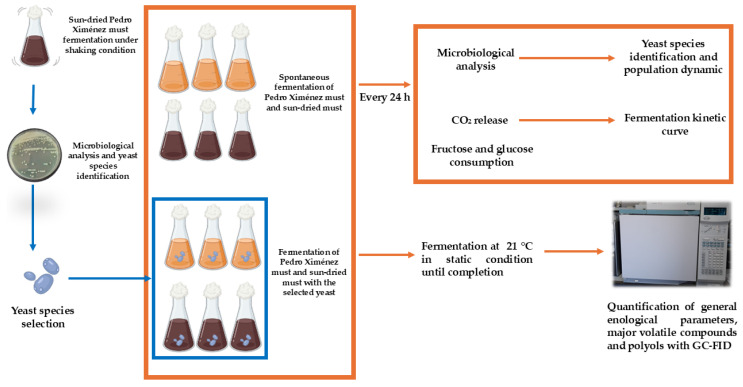
Workflow of material and methods/experimental design. GC-FID: gas chromatography with FID detector. Created with BioRender.com.

The workflow for the Materials and Methods section is detailed in Figure 1.

### 2.1. Isolation and Identification of Yeasts from Sun-Dried Pedro Ximénez Must Spontaneous Fermentation

For the isolation of potentially fructophilic yeasts, a sun-dried grape must of the Pedro Ximénez variety, kindly provided by Bodegas San Acacio (Montilla–Moriles PDO), with a sugar content of 450 g/L was employed. A total of 150 mL of sun-dried grape must in a 250 mL sterile Erlenmeyer flask was incubated at 28 °C and 135 RPM for 14 days and was sampled on days 0, 2, 7, and 14. Samples were seeded in WL (OXOID CM 0501; Hampshire, UK) agar medium (50 g/L dextrose, 4 g/L yeast extract, 5 g/L tryptone, 0.022 g/L bromocresol green, and 20 g/L agar) and incubated for 72 h at 28 °C. Each sample was cultured in duplicate and 10 colonies per plate were randomly isolated in axenic cultures on YPD agar (10 g/L yeast extract, 20 g/L peptone, 20 g/L dextrose, and 20 g/L agar). A total of 60 colonies were obtained.

### 2.2. Identification and Characterization of Yeast Isolates

Isolated yeasts were identified by Matrix-Assisted Laser Desorption/Ionization Time of Flight (MALDI-TOF) Mass Spectrometry with MALDI-TOF/TOF “ULTRAFLEXTREME” (Bruker Daltonics, Bremen, Germany) equipment. The generated spectra were processed using MALDI Biotyper compass (MBT Compass; Bruker, Billerica, MA, USA) software version 4.1.1, which calibrates the spectra and automates measurement and identification procedures prior to result matching. The resulting spectra were then compared to reference profiles in the MBT Compass Library (Bruker) as described in Carbonero-Pacheco [15]. Subsequently the isolates were inoculated in triplicate on *β*-glucosidase detection agar, (5 g/L arbutin (Sigma-Aldrich; St. Louis, MO, USA), 1 g/L yeast extract, 20 g/L agar and 0.2% of a 1% (*w*/*v*) iron chloride solution). Plates were incubated for 15 days at 28 °C. Dark black cultures were considered positive [16].

To measure the glucose and fructose consumption rate and evaluate the fructophilic feature of the identified species, 3 random isolates of each species were inoculated separately with an initial yeast population of 5 × 10^6^ cells/mL in sterile 100 mL Erlenmeyer flasks containing 100 mL of synthetic must (10 g/L yeast extract, 20 g/L peptone, 200 g/L dextrose, and 200 g/L fructose), adjusted to pH 3.5 by the addition of tartaric acid (Sigma-Aldrich; St. Louis, MO, USA). Each isolate was incubated at 21 °C and 70% relative humidity (RH) under static conditions in duplicate for 6 days. Glucose and fructose content after the trials was measured using a commercial test kit following manufacturer’s instructions (D-Fructose/D-Glucose Assay Kit, Megazyme, Bray, Ireland). Yeast isolates with the highest fructose/glucose ratio consumption were selected for further experiments with grape must.

### 2.3. Must Fermentation Conditions

The fresh and dried grape musts of the Pedro Ximénez variety (Montilla–Moriles PDO), harvested in 2023 came from the Pérez Barquero SA and San Acacio wineries, respectively, with a sugar content of 210 g/L for the fresh and 500 g/L for the dried must. Three conditions, J15, J33 and SF, were established for each must to assess the impact of the two selected strains, *Zygosaccharomyces rouxii*, *Starmerella lactis-condensi* and the spontaneous fermentation with the indigenous microbiota respectively.

For inocula preparation, selected strains were previously pre-cultured in the synthetic must described in Section 2.2. An initial yeast population of 5 × 10^6^ cells/mL was inoculated in each case.

For each condition, three biological replicates with 900 mL of grape must at a pH of 3.5 (adjusted by the addition of tartaric acid) in sterile 1 L Erlenmeyer flasks were conducted; a temperature of 21 °C and 70% relative humidity (RH) under static conditions was maintained until the end of alcoholic fermentation (weight loss due to CO_2_ release less than 1 g/day). During the fermentation process, every 24 h, each replicate was measured on a scale to register the mass loss due to CO_2_ release and 5 mL was sampled for microbiological and glucose/fructose consumption analysis.

### 2.4. Measurement of Enological Parameters

Chemical analyses (ethanol content, pH, titratable acidity, free and total sulfur dioxide (SO_2_), volatile acidity, and glycerol) were performed according to the recommendations and protocols of the International Organization of Vine and Wine (OIV) [17]; however, the most significant aspects of these procedures are described below. A Crison GLP 21 + pH meter was employed to determine pH. Titratable acidity was measured by titrating the wine to pH 7.0 with a standard sodium hydroxide solution (NaOH). Free and total SO_2_ by amperometric titration with iodine. An Alcolyzer 3001 alcohometer (Anton Paar; Graz, Austria) was used to analyze the ethanol content. The quantification of acetic acid and glycerol content (g/L) was performed with the Y15 chemical analyzer, using an absorbance of 500 nm (Biosystems; Barcelona, Spain).

### 2.5. Quantification of Major Volatile Aroma Compounds and Polyols

To analyze the major volatile compounds (concentration > 10 mg/L) in wine, an Agilent 6890 gas chromatograph (Santa Clara, CA, USA) was employed. These compounds evaporate at room temperature and play a significant role in the sensorial profile of wine. The gas chromatography system is equipped with a flame ionization detector (FID) and a specialized “CP-Wax 57 CB” column (60 m length × 0.25 mm internal diameter × 0.2 μm film thickness) optimized for this application. Sample injection volume was 0.7 μL per replicate, with a total elution time of 80 min for the target volatile compounds and polyols [18]. A total of 10 mL of wine samples was prepared by adding 1 mL of internal standard solution (1.018 g/L 4-methyl-2-pentanol) in a 14% (*v*/*v*) ethanol solution alongside 0.2 g of calcium carbonate. The mixture was sonicated for 30 s and then centrifuged at 5000 rpm (10 min, 2 °C) to remove tartaric acid from the wine and the supernatant from this process was injected for analysis. The absolute quantification of methanol, higher alcohols (1-propanol, isobutanol, isoamyl alcohol, and 2-phenylethanol), 1,1-diethoxyethane, acetaldehyde, acetoin, ethyl acetate, ethyl lactate, diethyl succinate, and the polyol 2,3-butanodiol (levo and meso forms) was performed using a calibration table built with the standard solutions from Thermo Fisher Scientific (Waltham, MA, USA), Merck (Darmstadt, Germany), and Sigma-Aldrich (St. Louis, MO, USA), containing a known concentration of the compounds. A total of 1 mL of the internal standard solution was added to 10 mL of each standard solution samples, the resulting mixtures were homogenized prior to injection.

### 2.6. Microbiological Analysis

Samples obtained during alcoholic fermentation of grape musts were cultured by duplicate in WL agar medium and incubated at 28 °C for 72 h; subsequently, the plates were incubated at 10 °C for 120 h to allow colonies full growth and facilitate colony isolation. Ten random colonies of each agar plate were identified by MALDI-TOF MS.

### 2.7. Statistical Analyses

Data presented in Tables and Figures are the average values of a minimum of three biological replicates, each analyzed in triplicate for every studied condition. Statistical analysis was performed using multiple comparison analysis (MCA) for each chemical parameter using the Bonferroni’s test at a confidence level of 95% (i.e., a = 0.05 significance level to identify those variables showing significant differences in the wine samples). MCA categorizes samples with significant differences into homogeneous groups (HG). Averages with different HGs show statistically significant differences at the 95.0% confidence level. To differentiate between averages, Fisher’s least significant difference (LSD) procedure was conducted. Principal Component Analysis (PCA) was employed to reduce the dimensionality of these data and identify the most significant variables in the samples. The results were visualized using a biplot, which graphically represents both the samples and variables. Groups were statistically analyzed to determine the fermentation condition (SF, J15 and J33).

## 3. Results

This section presents the results related to the selection of fructophilic yeast strains in synthetic media and their subsequent performance in natural grape musts.

### 3.1. Isolation, Identification, and Characterization of Yeast from Sun-Dried Grape Must

During the isolation and identification work on samples coming from sun-dried grape must fermentation, only six different yeast species were identified among all the grown colonies, all of them non-*Saccharomyces* (*Hanseniaspora opuntiae*, *Lachancea thermotolerans*, *Pichia kudriavzevii*, *Starmerella lactis-condensi*, *Torulaspora delbrueckii*, and *Zygosaccharomyces rouxii*) (Table 1). Species succession was observed during the different sampling times: at T_0_, most species were found, with *H. opuntiae* being the most abundant, while *S. lactis-condensi* could not be isolated at the beginning of the fermentation, likely due to its low concentration. It is, together with *Z. rouxii*, one of the only species identified in the final sampling time (T_14_).

None of the identified isolates exhibited β-glucosidase activity which could indicate a limitation in aroma precursor hydrolysis. However, differences were observed in the glucose and fructose consumption in synthetic medium. Table 2 shows the mean of the glucose and fructose consumption (%) of each identified species; *H. opuntiae*, *S. lactis-condensi*, *T. delbrueckii*, and *Z. rouxii* show high percentages of fructose consumption in synthetic medium after 6 days of incubation, but *T. delbrueckii* also consumed a significant percentage of glucose. Therefore, considering the highest percentages of fructose consumption, two strains—*S. lactis-condensi* and *Z. rouxii*—were selected for the alcoholic fermentation experiments using natural musts.

### 3.2. Inoculation of Selected Yeasts in Fresh and Sun-Dried Grape Must

#### 3.2.1. Alcoholic Fermentation Rates and Sugar Consumption

**Figure 2:**
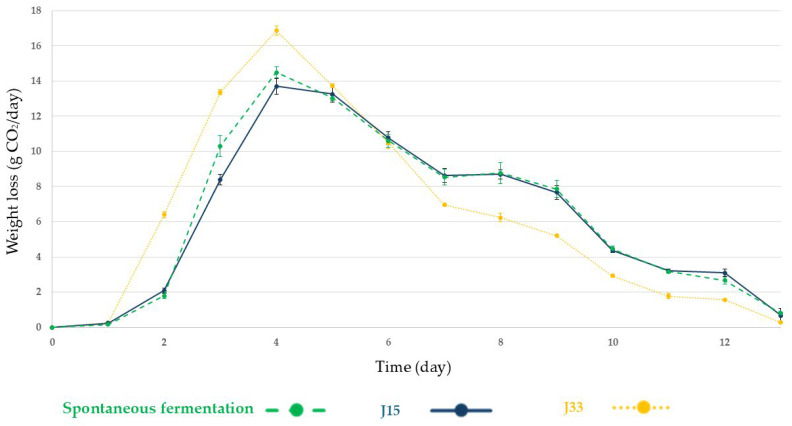
Fermentation evolution by CO_2_ production and release during alcoholic fermentation of fresh grape must under the tested conditions. Spontaneous fermentation: green dashed line; J15, experiment inoculated with *Z. rouxii*: blue line; J33, experiment inoculated with *S. lactis-condensi*: yellow dotted line. Error bars represent the standard deviation of the averages from three biological replicates.

**Figure 3:**
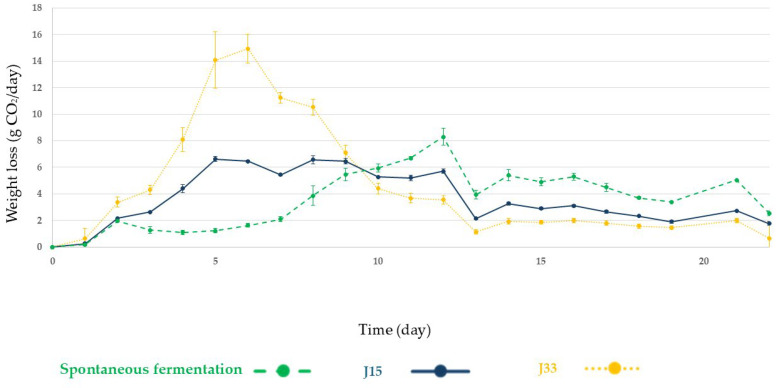
Fermentation evolution by CO_2_ production and release during alcoholic fermentation of sun-dried grape must under the tested conditions. Spontaneous fermentation: green dashed line; J15, experiment inoculated with *Z. rouxii*: blue line; J33, experiment inoculated with *S. lactis-condensi*: yellow dotted line. Error bars represent the standard deviation of the averages from three biological replicates.

Figure 2 and Figure 3 show the mass loss evolution due to CO_2_ release during the alcoholic fermentation of the tested conditions in fresh grape must and sun-dried grape must respectively. In fresh grape must, all fermentations follow a similar profile with the largest CO_2_ release occurring on day 4, with J33 being the one with the highest mass loss and J15 the lowest. Alcoholic fermentation was completed on day 13 in the three studied conditions.

In sun-dried grape must (Figure 3) fermentation profiles vary depending on the condition studied, with the largest CO_2_ release occurring on days 6, 8, and 13 for the J33, J15, and SF conditions, respectively. It can be noted that most of the mass loss due to CO_2_ release in the J33 condition was recorded between days 4 and 9, while this phenomenon occurred gradually during the whole process for J15 and SF conditions until alcoholic fermentation was completed on day 22.

**Figure 4:**
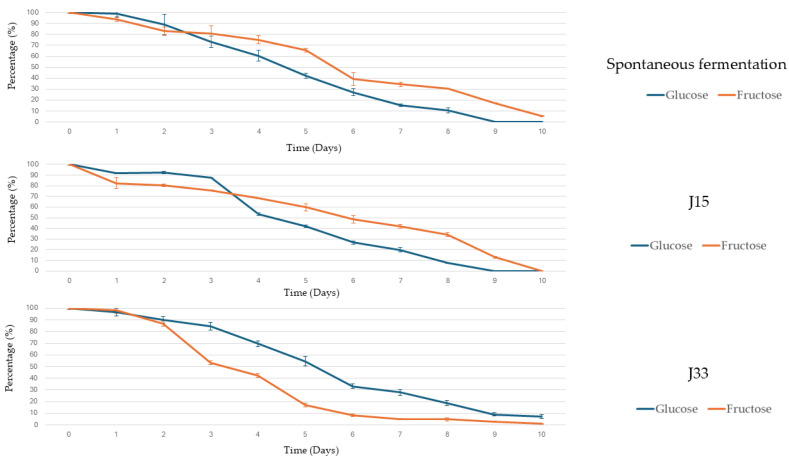
Glucose and fructose consumption (%) under each condition during fresh grape must alcoholic fermentation. Error bars represent the standard deviation of the averages from three biological replicates.

**Figure 5:**
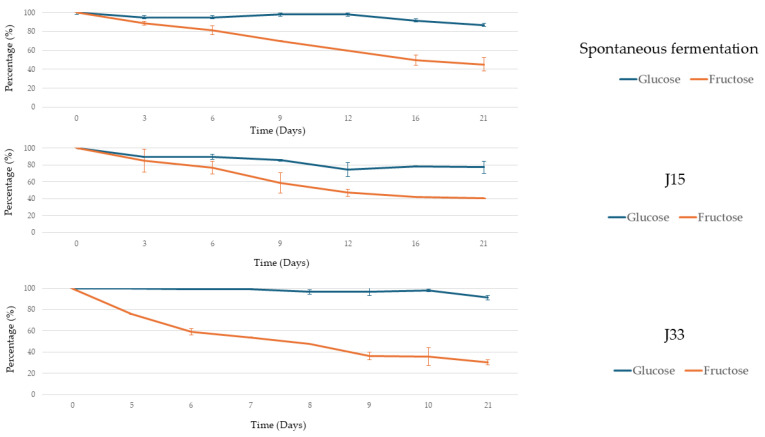
Glucose and fructose consumption (%) under each condition during sun-dried grape must alcoholic fermentation. Error bars represent the standard deviation of the averages from three biological replicates.

Figure 4 illustrates the evolution of sugar consumption in fresh grape must. This figure shows that fructose was consumed preferentially under the J33 condition, whereas glucose was consumed at a higher rate under the other two conditions. In all cases, the microbiota present in the must was able to consume all available sugars, which were depleted by the tenth day. In contrast, when analyzing the data obtained from sun-dried grape must (Figure 5), fructose is preferentially consumed in all cases. However, neither glucose nor fructose is completely depleted; J33 showed the highest percentage of fructose consumption in the shortest period of time.

#### 3.2.2. Microbiological Analysis

**Figure 6:**
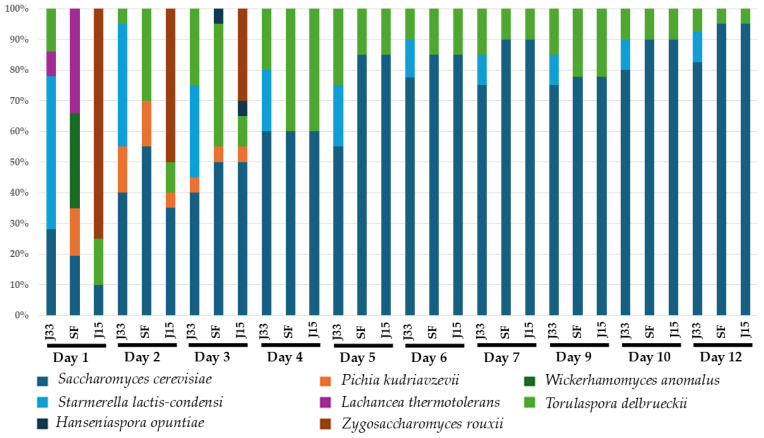
Relative abundance (%) of isolated and identified species during alcoholic fermentation of fresh grape must. J33: fermentation inoculated with *S. lactis-condensi*; J15: fermentation inoculated with *Z. rouxii*; SF: spontaneous fermentation with indigenous microbiota.

**Figure 7:**
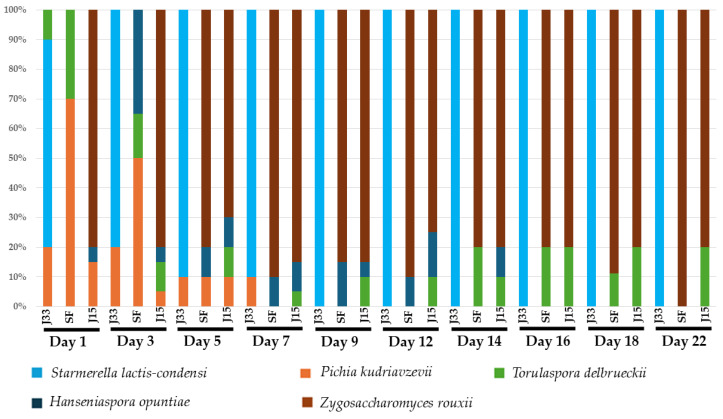
Relative abundance (%) of isolated and identified species during alcoholic fermentation of Pedro Ximénez sun-dried grape must. J33: fermentation inoculated with *S. lactis-condensi*; J15: fermentation inoculated with *Z. rouxii*; SF: spontaneous fermentation with indigenous microbiota.

Yeast identification reveals the presence of 8 and 5 different species during alcoholic fermentation of fresh must and sun-dried must respectively. Figure 6 and Figure 7 show the species dynamics in all conditions, with *S. cerevisiae* being the most abundant yeast species from day 4 onwards in the fresh must, regardless of the inoculum used. *S. lactis-condensi* was identified only in the J33 condition, where it was inoculated, and persisted throughout the entire fermentation process. In contrast, *Z. rouxii*, which was inoculated in the J15 condition, was isolated only up to the third day of fermentation, after which it was replaced by *S. cerevisiae* and *T. delbrueckii* until the end of alcoholic fermentation. Conversely, in the sun-dried must (Figure 7), *Z. rouxii* was the dominant species in the SF and J15 conditions, whereas *S. lactis-condensi* was predominant in J33 condition, where no other species were detected in any replicate (*n* = 3) beyond day 9.

#### 3.2.3. Enological Parameters and Major Aroma Compounds and Polyols

Table 3 and Table 4 summarize the 23 variables analyzed in the wines obtained after fermentation of Pedro Ximénez grape must and sun-dried grape must, respectively. Of these 23 variables, 14 were measured by GC-FID and 9 according to OIV methods. Diethoxyethane was not detected under any of the experimental conditions in either must.

**Figure 8:**
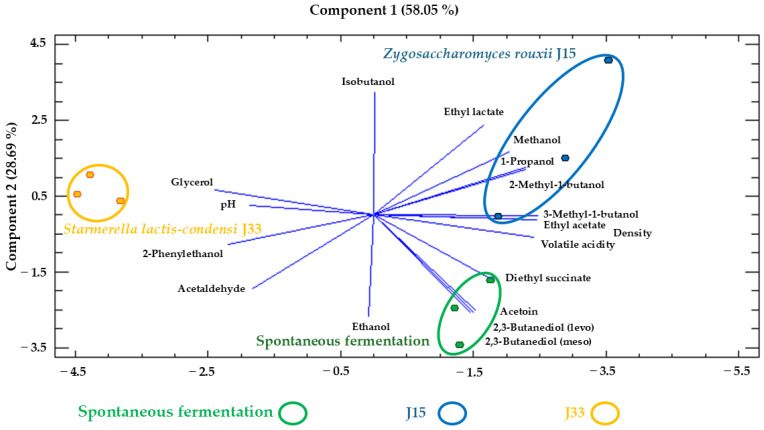
Principal component analysis (PCA) of wines obtained in the alcoholic fermentation of Pedro Ximénez grape must with different strategies. The analysis was carried out with the oenological parameters, major volatile compounds, and polyols studied. J33: wine obtained from the experiment inoculated with *S. lactis-condensi*; J15: wine obtained from the experiment with *Z. rouxii*; SF: wine obtained from spontaneous fermentation with indigenous microbiota.

In wines resulting from the alcoholic fermentation of fresh grape must, the MCA test shows significant differences in 8 of the variables analyzed among the three conditions studied. Moreover, all variables except for free SO_2_ showed significant differences between at least two conditions. The results obtained for the SF and J15 conditions were more similar to each other, whereas J33 differed more markedly in its composition. Figure 8 presents these differences in a PCA, where the sum of components 1 and 2 accounts for 86.74% of the total variance. In the biplot, samples from the J33 condition are positioned on the left, being strongly influenced by glycerol, pH, 2-phenylethanol, and acetaldehyde, whereas those from J15 and SF are located on the right, influenced by the remaining variables except for isobutanol and ethanol.

**Figure 9:**
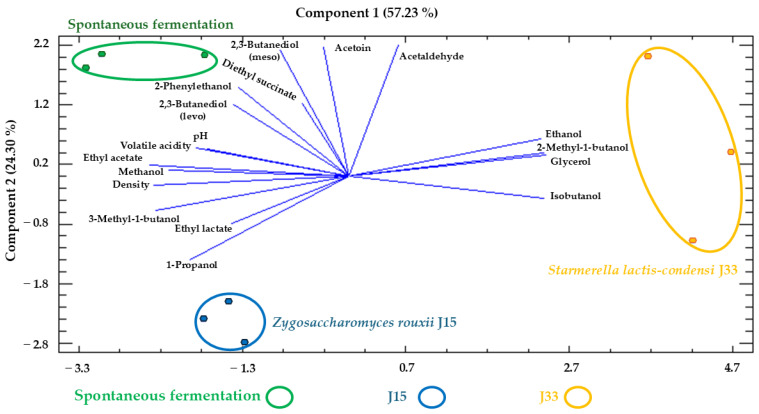
Principal component analysis (PCA) of wines obtained in the alcoholic fermentation of Pedro Ximénez sun-dried grape must with different strategies. The analysis was carried out with the oenological parameters, major volatile compounds, and polyols studied. J33: wine obtained from the experiment inoculated with *S. lactis-condensi*; J15: wine obtained from the experiment inoculated with *Z. rouxii*; SF: wine obtained from spontaneous fermentation with indigenous microbiota.

Analyzing the results of the alcoholic fermentation of sun-dried grape must, significant differences were observed among the three conditions in 5 out of the 23 variables, and two distinct groups were identified in 10 of the remaining 17 variables (Table 4). As in fresh grape must, the J33 condition differs from J15 and SF. Figure 9 illustrates these differences in a PCA, where the components together account for 81.53% of the total variance. In this plot, samples from the J33 condition are located on the right, mainly influenced by ethanol, 2-methyl-1-butanol, glycerol, and isobutanol, whereas J15 and SF are affected by multiple variables, especially by ethyl acetate, methanol, and density.

## 4. Discussion

The use of selected yeasts, generally strains from the *S. cerevisiae* species, as starters is a common practice in alcoholic fermentation processes due to their high fermentative power, which allows them to consume all available sugars in the media [20,21]. However, the production of vinegar containing gluconic acid involves the use of fructophilic yeasts in order to leave some of the glucose in the original medium unconsumed so that this sugar can be converted into gluconic acid at a later stage.

During the isolation and selection of yeast from sun-dried grape must, a succession of yeast species was observed in which *S*. *lactis-condensi* and *Z. rouxii*—described by other authors as osmophilic—prevail, while other species, such as *H*. *opuntiae*, which belongs to the main genus present in ripe grapes, disappear [9,22,23]. In the sugar consumption test in synthetic must, strains of these three species showed a fructophilic trend, however, the low fermentative power of the isolated *H. opuntiae* strains made them unsuitable for further trials in natural musts (Table 2).

In contrast, *S. lactis-condensi* and *Z. rouxii* strains, evaluated in two musts of the Pedro Ximénez grape variety with different sugar concentrations, were selected due to their fructophilic behavior. Firstly, in the fresh grape must, both yeasts have a low impact on the profiles of alcoholic fermentation (Figure 2), even with a high initial inoculum. Comparing the fermentation evolution with the relative abundance of species (Figure 6), the highest CO_2_ release coincided with the dominance of *S. cerevisiae* in all conditions which indicates the impact of the indigenous microbiota in the fermentation process. However, the glucose and fructose consumption data (Figure 4) indicate that, in the J33 condition, *S. lactis-condensi* remains present despite being outcompeted by the indigenous microbiota. The fructophilic nature of *S. lactis-condensi* had already been observed during the selection of strains isolated from sun-dried must, and has also been reported in other studies, highlighting not only its ability to metabolize this sugar but also its capacity to produce a vigorous alcoholic fermentation [24,25]. However, under the J15 condition, where *Z. rouxii* was inoculated, glucose was consumed faster than fructose, despite this species being described by other authors as both fructophilic and osmophilic [26]. This may be due to the selected strains’ inability to compete with better-adapted, highly fermentative indigenous microbiota, resulting in their disappearance after day 3 and displaying a pattern very similar to the spontaneous fermentation (SF) in terms of fermentation evolution, sugar consumption, and yeast population dynamics (Figure 2, Figure 4 and Figure 6, respectively). These data indicate that, although *Z. rouxii* is considered a spoilage yeast in the industry due to its ability to survive in environments with high sugar concentrations and low water activity [27], this species is outcompeted and displaced mostly by *S. cerevisiae* or *T. delbrueckii* in fresh must during the first stages of the alcoholic fermentation (Figure 6).

Analyzing the wine composition resulting from the alcoholic fermentation of fresh grape must (Table 3 and Figure 8), the J33 condition stands out due to its lower content of higher alcohols (1-propanol, isobutanol, 2-methyl- and 3-methyl-1-butanol, and 2-phenylethanol) and 2,3-butanediol, as well as a higher concentration of glycerol compared to the J15 and SF conditions, which are very similar to each other. It is well known that the production of these volatile compounds is directly related to the yeast population present during the alcoholic fermentation process [28]. High glycerol and low acetic acid production by *S. lactis-condensi* has been confirmed in different fermentative processes such as beer and wine production [24,29], and the lower concentration of higher alcohols and 2,3-butanediol could be related to the distinctive metabolism of the *Starmerella* genus. Other studies have shown that *S. bombicola* exerts a certain influence on the expression of key enzymes involved in the production of these compounds, such as pyruvate decarboxylase (Pdc1) and alcohol dehydrogenase (Adh1) by *S. cerevisiae* during the mixed fermentation of synthetic must [30]. This could explain the differences observed between conditions; however, further research with this specific species is necessary to clarify the reasons for these differences.

The use of *S. lactis-condensi* in fresh must influences the alcoholic fermentation process and the final composition of the resulting wine, with fructose being consumed before glucose. However, due to the presence of an autochthonous microbiota with high fermentative capacity, the result is a product without glucose, which would not be suitable as a base for vinegar with gluconic acid content. Therefore, it is necessary to optimize the use of *S. lactis-condensi* in this type of substrate through alternative strategies that reduce the impact of other microorganisms present in the must. Such strategies could include the application of sterilization techniques prior to the inoculation of the fructophilic yeast, such as pasteurization or chemical sterilization with dimethyl dicarbonate (DMDC) [31].

In sun-dried grape must, the inoculation with the selected yeasts has a greater influence in the fermentation profiles than in the fresh must. In Figure 3 could be observed a faster and higher CO_2_ production in J33 condition than the registered in J15 and SF in the first ten days of alcoholic fermentation. This is consistent with the observations regarding sugar consumption (Figure 5), where, at this point, the yeast in the J33 condition has consumed approximately 50% of the available fructose and a low percentage of glucose, with *S. lactis-condensi* dominating the entire process (Figure 7). These data highlight the osmotolerant and fructophilic nature of *S. lactis-condensi*, and are in agreement with previous reports by other authors who have abundantly isolated this species from environments with high sugar concentrations, such as Essence, Manna, and the “mothers” of vino cotto (cooked wine), demonstrating its enhanced growth when consuming fructose [9,25,32]. Complete sugar depletion was not achieved under any of the studied conditions, this could be due to osmotic pressure combined with ethanol production which leads to a fermentation rate decrease. In other natural concentrated must fermentations such as Essence, fermentation could last for years due to osmotic pressure [9,33]. However, this phenomenon is of interest in the scope of this work because it allows for the production of a wine base containing ethanol and glucose. The results obtained for J15 and SF are similar, with CO_2_ release occurring somewhat more rapidly in the former. The low initial CO_2_ release in SF is due to the lower initial concentration and diversity of yeasts in this must compared to fresh must. It is well known that the microbiota associated with grapes changes during the ripening process, which alters water availability and the sugar gradient. Furthermore, the “asoleo” process, characteristic of the Montilla–Moriles PDO, intensifies this environmental pressure [15,34,35].

Table 4 shows that the wine obtained in the J33 condition, with an *S. lactis-condensi* inoculum, has a higher ethanol content (11.10 ± 0.15) than the J15 and SF conditions (8.06 ± 0.07 and 8.48 ± 0.19, respectively). In addition to ethanol, a high production of glycerol is also observed in both fresh and sun-dried must, which would indicate that *S. lactis-condensi* is a good producer of this metabolite. This high glycerol yield is associated with the *Starmerella* genus; due to their osmophilic nature, these yeasts tend to produce and accumulate glycerol (as a compatible solute) to prevent dehydration by balancing intracellular osmolarity, allowing them to thrive in high-sugar environments. This could explain the higher fermentation rate observed in the J33 condition in sun-dried grape must [36,37]. Lower concentrations of ethyl acetate and volatile acidity in this condition may be related to reduced stress in the yeast population. Indeed, other authors who have carried out alcoholic fermentations in musts with high sugar concentrations using other yeasts, such as *S. cerevisiae* or *L. thermotolerans*, have reported high concentrations of these metabolites, which, at elevated concentrations, could cause defects in the resulting wine [14,38].

## 5. Conclusions

The sun-drying process (“asoleo”) carried out in the Montilla–Moriles PDO allows the presence of a mixture of non-conventional yeasts, all of them non-*Saccharomyces* (*Hanseniaspora opuntiae*, *Lachancea thermotolerans*, *Pichia kudriavzevii*, *Starmerella lactis-condensi*, *Torulaspora delbrueckii*, and *Zygosaccharomyces rouxii*). Among them, because of their properties regarding fructophilic behavior, *S. lactis-condensi* and *Z. rouxii* were selected for carrying out alcoholic fermentations in grape musts, both fresh and sun-dried concentrated ones, for converting fructose and glucose into ethanol but leaving part of the initial glucose unconsumed.

The use of the *S. lactis-condensi* strain in fresh must, although it leads to the depletion of fructose prior to glucose, requires technical improvements to prevent the exhaustion of the total sugar content, such as the application of heat or chemical sterilization. In sun-dried grape must, the *S. lactis-condensi* strain could be employed for the production of base wines with high glucose content, suitable for vinegar production with gluconic acid, due to its ability to generate high ethanol levels without consuming glucose.

On the other hand, the use of *S. lactis-condensi*, because of the low volatile acidity and high glycerol production during fermentation, might be of industrial interest for the elaboration of young wines from this type of must.

The strain *Z. rouxii* has not successfully established itself in fresh must, and its use in sun-dried must does not result in significant differences compared to SF in the resulting wine.

Based on the results obtained, we conclude that the inoculation of Pedro Ximénez sun-dried must with *S. lactis-condensi* represents a promising candidate in the enology field due to its osmophilic and fructophilic features, allowing an alcoholic fermentation that leaves glucose unfermented, which can be used, for instance, to produce other products such as vinegars containing gluconic acid.

## Figures and Tables

**Table 1:** Relative abundance (%) of yeast species identified at different sampling times in the sun-dried grape must spontaneous fermentation with shaking. T_0_: before spontaneous fermentation; T_2_: day two; T_7_: day seven; T_14_: day fourteen.

Species	T_0_	T_2_	T_7_	T_14_
*Hanseniaspora opuntiae*	64.23	0.00	0.00	0.00
*Lachancea thermotolerans*	21.02	9.20	2.30	0.00
*Pichia kudriavzevii*	2.90	0.00	0.00	0.00
*Starmerella lactis-condensi*	0.00	0.00	1.40	74.76
*Torulaspora delbrueckii*	7.43	3.16	0.00	0.00
*Zygosaccharomyces rouxii*	4.43	87.65	96.30	25.25

**Table 2:** Mean of glucose and fructose consumption (%) ± standard deviation for each yeast species after 6 days of incubation in synthetic medium.

Species	Glucose	Fructose
*Hanseniaspora opuntiae*	13.92 ± 2.64	20.96 ± 6.45
*Lachancea thermotolerans*	23.00 ± 4.19	18.59 ± 1.56
*Pichia kudriavzevii*	9.15 ± 0.91	8.25 ± 0.35
*Starmerella lactis-condensi*	26.32 ± 3.95	58.95 ± 6.92
*Torulaspora delbrueckii*	36.42 ± 1.21	29.76 ± 0.08
*Zygosaccharomyces rouxii*	28.76 ± 7.47	56.27 ± 4.09

**Table 3:** General oenological parameters and major volatile compounds and polyols detected in the fermented fresh grape must. CAS: identification number assigned by the Chemical Abstracts Service; OT: odor threshold; J33: wine obtained from the experiment inoculated with *S. lactis-condensi*; J15: wine obtained from the experiment inoculated with *Z. rouxii*; SF: wine obtained from spontaneous fermentation with indigenous microbiota. Values are reported as the mean ± standard deviation. The different letters (abc) indicate homogeneous groups that significantly differ statistically in the parameters between wines (*p* < 0.05, F-test). OT was obtained from Carbonero-Pacheco et al. [19].

Compound	Method of Detection	CAS	OT (mg/L)	Odor/Flavor Description	J33	J15	SF
	**Parameters measured according OIV**						
Ethanol (% *v*/*v*)		64-17-5	10	Alcoholic	11.88 ± 0.05 ^a^	11.74 ± 0.17 ^a^	12.12 ± 0.05 ^b^
Glycerol (mg/L)		56-81-5	-	Confers body and smoothness and a sweet taste	8070 ± 34.00 ^b^	4890 ± 25.00 ^a^	5030 ± 19.00 ^b^
pH		-	-	-	3.17 ± 0.01 ^b^	3.09 ± 0.03 ^a^	3.11 ± 0.06 ^ab^
Density		-	-	-	0.9897 ± 0.0002 ^a^	0.9936 ± 0.0002 ^b^	0.9936 ± 0.0003 ^b^
Volatile acidity (g/L)		64-19-7	200	Vinegar	0.11 ± 0.02 ^a^	0.74 ± 0.06 ^b^	0.74 ± 0.05 ^b^
Titratable acidity (g/L)		-	-	-	7.08 ± 0.13 ^a^	9.08 ± 0.44 ^a^	8.69 ± 0.37 ^b^
Free SO_2_ (mg/L)		-	-	-	8.30 ± 1.15 ^a^	7.30 ± 0.47 ^a^	8.30 ± 0.47 ^a^
Total SO_2_ (mg/L)		-	-	-	31.33 ± 1.15 ^a^	38.30 ± 1.52 ^b^	39.66 ± 0.57 ^b^
Total extract (g/L)		-	-	-	21.86 ± 0.25 ^b^	20.62 ± 0.19 ^a^	21.42 ± 0.07 ^b^
	**GC-FID**						
Acetaldehyde (mg/L)		75-07-0	10	Over-ripe apple	56.98 ± 0.62 ^b^	50.75 ± 3.88 ^a^	55.20 ± 0.70 ^ab^
Ethyl acetate (mg/L)		141-78-6	7.5	Pineapple, varnish, balsamic	28.27 ± 0.02 ^a^	331.19 ± 3.28 ^c^	262.87 ± 1.72 ^b^
1,1-Diethoxyethane (mg/L)		105-57-7	1	Refreshing, pleasant, fruity-green	0.00 ± 0.00	0.00 ± 0.00	0.00 ± 0.00
Methanol (mg/L)		67-56-1	668	Chemical, medicinal	63.73 ± 2.60 ^a^	78.43 ± 6.47 ^b^	69.66 ± 4.60 ^ab^
1-Propanol (mg/L)		71-23-8	830	Ripe fruit, alcohol	28.20 ± 0.28 ^a^	35.18 ± 2.31 ^c^	31.85 ± 0.95 ^b^
Isobutanol (mg/L)		78-83-1	40	Alcohol, wine like, nail polish	57.45 ± 0.82 ^b^	59.79 ± 2.08 ^b^	52.16 ± 0,83 ^a^
2-Methyl-1-butanol (mg/L)		137-32-6	30	N.f.	30.67 ± 0.07 ^a^	33.62 ± 1.10 ^c^	32.26 ± 0.21 ^b^
3-Methyl-1-butanol (mg/L)		123-51-3	30	Alcohol, nail polish	124.45 ± 1.51 ^a^	194.54 ± 6.46 ^c^	180.98 ± 2.35 ^b^
Acetoin (mg/L)		513-86-0	30	Buttery, creamy	37.10 ± 6.79 ^ab^	52.79 ± 4.68 ^b^	76.37 ± 8.89 ^c^
Ethyl lactate (mg/L)		97-64-3	7.5	Strawberry, raspberry, buttery	15.16 ± 0.05 ^a^	15.61 ± 0.28 ^b^	15.21 ± 0.03 ^a^
2,3-butanediol (levo) (mg/L)		24347-58-8	668	Buttery, creamy	293.24 ± 16.73 ^a^	390.33 ± 46.84 ^b^	524.71 ± 67.42 ^c^
2,3-butanediol (meso) (mg/L)		5341-95-7	668	Buttery, creamy	66.70 ± 7.61 ^a^	102.15 ± 21.33 ^ab^	146.35 ± 27.32 ^b^
Diethyl succinate (mg/L)		123-25-1	100	Over-ripe, lavender	17.64 ± 0.27 ^b^	10.45 ± 1.90 ^a^	11.00 ± 0.28 ^a^
2-Phenylethanol (mg/L)		60-12-8	10	Floral	4.85 ± 0.27 ^a^	3.26 ± 0.20 ^b^	3.83 ± 0.66 ^a^

**Table 4:** General oenological parameters and major volatile compounds and polyols detected in the fermented sun-dried grape must. CAS: identification number assigned by the Chemical Abstracts Service; OT: odor threshold; J33: wine obtained from the experiment inoculated with *S. lactis-condensi*; J15: wine obtained from the experiment inoculated with *Z. rouxii*; SF: wine obtained from spontaneous fermentation with indigenous microbiota. Values are reported as the mean ± standard deviation. The different letters (abc) indicate homogeneous groups that significantly differ statistically in the parameters between wines (*p* < 0.05, F-test). OT was obtained from Carbonero-Pacheco et al. [19].

Compound	Method of Detection	CAS	OT (mg/L)	Odor/Flavor Description	J33	J15	SF
	**Parameters measured according OIV**						
Ethanol (% *v*/*v*)		64-17-5	10	Alcoholic	11.10 ± 0.15 ^c^	8.06 ± 0.07 ^a^	8.48 ± 0.19 ^b^
Glycerol (mg/L)		56-81-5	-	Confers body and smoothness and a sweet taste	9950 ± 93.00 ^b^	4730 ± 90.00 ^a^	4950 ± 30.00 ^a^
pH		-	-	-	3.47 ± 0.01 ^b^	3.51 ± 0.03 ^a^	3.53 ± 0.06 ^a^
Density		-	-	-	1.130 ± 0.004 ^a^	1.145 ± 0.005 ^b^	1.147 ± 0.005 ^b^
Volatile acidity (g/L)		64-19-7	200	Vinegar	0.24 ± 0.04 ^a^	0.31 ± 0.03 ^ab^	0.36 ± 0.06 ^b^
Titratable acidity (g/L)		-	-	-	8.21 ± 0.31 ^a^	8.61 ± 0.15 ^a^	8.69 ± 0.34 ^a^
Free SO_2_ (mg/L)		-	-	-	9.33 ± 1.16 ^a^	10.67 ± 1.16 ^a^	10.67 ± 1.16 ^a^
Total SO_2_ (mg/L)		-	-	-	51.00 ± 3.46 ^a^	52.00 ± 1.73 ^a^	53.33 ± 0.58 ^a^
Total extract (g/L)		-	-	-	441.40 ± 0.57 ^c^	396.38 ± 0.60 ^b^	392.45 ± 0.56 ^a^
	**GC-FID**						
Acetaldehyde (mg/L)		75-07-0	10	Over-ripe apple	145.81 ± 15.38 ^b^	61.54 ± 6.61 ^a^	157.11 ± 7.92 ^b^
Ethyl acetate (mg/L)		141-78-6	7.5	Pineapple, varnish, balsamic	23.23 ± 0.37 ^a^	34.42 ± 0.16 ^b^	38.37 ± 0.44 ^c^
1,1-Diethoxyethane (mg/L)		105-57-7	1	Refreshing, pleasant, fruity-green	0.00 ± 0.00	0.00 ± 0.00	0.00 ± 0.00
Methanol (mg/L)		67-56-1	668	Chemical, medicinal	80.22 ± 16.85 ^a^	114.54 ± 2.26 ^b^	134.21 ± 1.83 ^b^
1-Propanol (mg/L)		71-23-8	830	Ripe fruit, alcohol	21.05 ± 1.07 ^a^	29.33 ± 0.33 ^c^	25.60 ± 0.18 ^b^
Isobutanol (mg/L)		78-83-1	40	Alcohol, wine like, nail polish	134.98 ± 0.76 ^c^	108.75 ± 0.05 ^b^	95.24 ± 0,67 ^a^
2-Methyl-1-butanol (mg/L)		137-32-6	30	N.f.	35.24 ± 0.41 ^b^	16.96 ± 0.20 ^a^	16.50 ± 0.85 ^a^
3-Methyl-1-butanol (mg/L)		123-51-3	30	Alcohol, nail polish	71.38 ± 1.60 ^a^	187.33 ± 0.42 ^c^	176.57 ± 0.93 ^b^
Acetoin (mg/L)		513-86-0	30	Buttery, creamy	103.76 ± 15.49 ^ab^	83.78 ± 1.77 ^a^	124.62 ± 13.19 ^b^
Ethyl lactate (mg/L)		97-64-3	7.5	Strawberry, raspberry, buttery	15.01 ± 0.12 ^a^	16.06 ± 0.44 ^a^	15.62 ± 1.10 ^a^
2,3-butanediol (levo) (mg/L)		24347-58-8	668	Buttery, creamy	159.60 ± 28.49 ^a^	170.04 ± 20.68 ^a^	215.31 ± 48.79 ^a^
2,3-butanediol (meso) (mg/L)		5341-95-7	668	Buttery, creamy	589.11 ± 58.68 ^a^	546.97 ± 37.06 ^a^	680.70 ± 13.96 ^b^
Diethyl succinate (mg/L)		123-25-1	100	Over-ripe, lavender	12.30 ± 1.50 ^a^	12.26 ± 0.94 ^a^	13.05 ± 0.48 ^a^
2-Phenylethanol (mg/L)		60-12-8	10	Floral	9.21 ± 0.75 ^a^	8.81 ± 0.25 ^a^	14.55 ± 3.51 ^b^

## Data Availability

The original contributions presented in this study are included in the article. Further inquiries can be directed to the corresponding author.
